# Exploring the effect of gestational diabetes mellitus on retinal vascular morphology by PKSEA-Net

**DOI:** 10.3389/fcell.2024.1532939

**Published:** 2025-01-08

**Authors:** Ligang Jiang, Yimei Ji, Mengting Liu, Ruolin Fang, Zhentao Zhu, Meizhen Zhang, Yuhua Tong

**Affiliations:** ^1^ Quzhou Aliated Hospital of Wenzhou Medical University, Quzhou People’s Hospital, Quzhou, Zhejiang, China; ^2^ Department of Ophthalmology, The Second Xiangya Hospital, Hunan Clinical Research Centre of Ophthalmic Disease, Central South University, Changsha, Hunan, China; ^3^ Second Clinical Medical College, Zhejiang Chinese Medical University, Hangzhou, Zhejiang, China; ^4^ Department of Ophthalmology, Huaian Hospital of Huaian, Huaian, Jiangsu, China; ^5^ Department of Obstetrics and Gynecology, Quzhou Kecheng People’s Hospital, Quzhou, Zhejiang, China

**Keywords:** gestational diabetes mellitus (GDM), retinal vascular analysis, retinal artery, retinal vein, artificial intelligence

## Abstract

**Background:**

Gestational diabetes mellitus (GDM) is a temporary metabolic disorder in which small retinal vessels may have experience subtle changes before clinical lesions of the fundus retina appear. An innovative artificial intelligence image processing technology was applied to locate and analyze the small retinal vessel morphology and accurately evaluate the changes of the small retinal vessels in GDM patients and pregnant women with normal blood glucose and non-pregnant women with normal blood glucose.

**Methods:**

The subjects were divided into three groups:GDM group, pregnant control group (PC), and normal control group (NC). Use optical coherence tomography angiography (OCTA) to collect OCT images of subjects,and perform quantitative identification and analysis of retinal vessel parameters based on artificial intelligence measurement software integrated the prior knowledge supervised edge-aware multi-task network (PKSEA-Net): Retinal arteriolar lumen diameter (RALD), retinal arteriolar outer diameter (RAOD), retinal venular lumen diameter (RVLD),retinal venular outer diameter (RVOD),arterial wall thickness (AWT),venular wall thickness (VWT),arterial wall to lumen ratio (AWLR),venular wall to lumen ratio (VWLR),arterial wall cross-sectional area (AWCSA),venular wall cross-sectional area (VWCSA), arteriovenous ratio (AVR).

**Results:**

This study revealed significant differences in RVOD, RVLD, VWT, VWCSA and AVR between the GDM group and the PC group (*p* = 0.005, *p* < 0.027, *p* = 0.008, *p* = 0.001, *p* = 0.022), significant differences in RVOD, RVLD, VWT, VWCSA and AVR between the GDM group and the NC group (*p* < 0.001, *p* = 0.001, *p* < 0.001, *p* < 0.001, *p* = 0.001). In GDM group, RVOD, RVLD, VWT and VWCSA increased, while AVR decreased. There were no significant differences in RVOD, RVLD, VWT, VWCSA and AVR between PC group and NC group (*p* = 0.139, *p* = 0.263, *p* = 0.107, *p* = 0.059, *p* = 0.218), and no significant differences in VWLR among the three groups (*p* > 0.05). No significant difference was observed in retinal artery vascular parameters (RAOD, RALD, AWT, AWLR, AWCSA) across the three groups (*p* > 0.05).

**Conclusion:**

There were increases in RVOD, RVLD, VWT, and VWCSA, decrease in AVR in patients with GDM. However, no significant difference of retinal vascular parameters was shown between normal pregnant women and normal non-pregnant women. PKSEA-Net can assist to identify changes in retinal vascular morphology and diagnose micro-vascular lesion early in normal pregnant women and high-risk groups of GDM.

## 1 Introduction

During pregnancy, the immune system of women experiences a series of intricate alterations. A dynamic equilibrium is sustained among inflammatory cytokines, which is of paramount significance for ensuring the normal development of the fetus. If this equilibrium is disturbed, it may give rise to pregnancy complications. Pregnant women with elevated blood glucose during pregnancy are prone to systemic complications (Soma-Pillay et al., 2016), which are closely related to changes in micro-circulation. This kind of clinical status is termed as “gestational carbohydrate metabolic disorder (CMDP),” which originates from traditional diabetes and can be classified into gestational diabetes mellitus (GDM) and preexisting diabetes (PexD) (Hadden and McLaughlin, 2009). GDM refers to diabetes that is first diagnosed during pregnancy, which is a variable state of hyperglycemia in the middle and late stages of gestation. The characteristics of diabetic retinopathy are typically indiscernible to the naked eye in GDM patients, and GDM serves as a risk factor for the development of type 2 diabetes mellitus (T2D) (Pota et al., 2024). Prior to the manifestation of clinical symptoms of diabetic retinopathy, subtle changes might have occurred in structure of the fundus retinal vessels. Meanwhile, GDM not only increases the risk of systemic complications for the mother but also raises potential risks for the fetus and newborn, such as macrosomia, fetal growth restriction, and neonatal respiratory distress syndrome, etc (Adam and Rheeder, 2017). Therefore, to reduce the incidence of GDM, we conducted in-depth research for GDM patients to figure out more subtle morphological changes of retinal vessels.

The emergence of Artificial Intelligence (AI) technology in the medical industry has developed into a dynamic and high-profile frontier research field, particularly in ophthalmology. The rapid development of AI has not only brought revolutionary changes in diagnosis and treatment of eye disease, but also made remarkable achievements in disease prediction and management (Yang et al., 2023; Ji et al., 2022; Zhang et al., 2022; Deng et al., 2024). Optical Coherence Tomography Angiography (OCTA) is an emerging technology that can non-invasively and quickly acquire OCT cross-sectional images of the retinal fundus with high resolution (Ma et al., 2024). In this study, we propose an artificial intelligence method called the prior knowledge supervised edge-aware multi-task network (PKSEA-Net), which aims to improve segmentation accuracy by enhancing the perception of edge information in retinal fundus OCT images. PKSEA-Net is capable of extracting fine vascular edge information to accomplish high-precision vascular parameter measurement. Additionally, it can enhance the process of feature extraction through the supervision of machine learning with external prior knowledge, achieving the purpose of extracting and calculating relevant medical parameter information. Previous research (Huang et al., 2024; Jiang et al., 2024) have demonstrated the scientificity and accuracy of this method, hence, we use this innovative technology to further study the changes of fundus retinal morphology in GDM population, providing a new perspective for early diagnosis and treatment.

## 2 Methods

### 2.1 General data and grouping

The study was approved by the Research Ethics Committee of Quzhou People’s Hospital and conducted in accordance with the tenets of the Declaration of Helsinki, with informed consent obtained from all the participants. All pregnant women were initially in treatment in the obstetrics department of Quzhou People’s Hospital and then referred to the ophthalmology department for examination.

This study included 160 participants who were divided into three experimental groups: 50 women with GDM as a GDM group, 55 pregnant women without diabetes as a pregnant control group (PC), and 55 non-pregnant women as a normal control group (NC) were included. All subjects underwent a comprehensive ophthalmic examination, encompassing Best Corrected Visual Acuity (BCVA), slit-lamp biomicroscopy, intraocular pressure, fundus photography, and OCTA, and none of them presented any ophthalmic or systemic diseases.

Enrolment criteria for all the experimental group were patients aged between 23 and 42 years old with good quality OCTA images. The specific criteria for each group were as follows: Pregnant women at least 24 weeks gestation who were diagnosed with GDM by the Oral Glucose Tolerance Test (OGTT) for GDM group, pregnant women with matching age and gestational age compared to GDM group who have normal blood glucose levels, and healthy, non-pregnant women with matching ages for NC group.

Exclusion criteria for the experimental subjects were as follows: a history of diabetes mellitus before pregnancy or diabetic nephropathy, hypertension (140/90 mmHg), autoimmune diseases, obesity, smoking, cardiovascular and cerebrovascular complications, diabetic macular edema, age-related macular degeneration, intraocular pressure (IOP) > 21 mmHg, glaucoma, optic nerve atrophy, uveitis, ocular inflammation, cataract, history of ocular surgery, intravitreal injections or trauma, and pregnant women with best-corrected visual acuity (BCVA) worse than 20/20 or refractive errors exceeding 3 diopters.

According to the standards of the Ministry of Health of the People’s Republic of China, a 75 g OGTT examination at 24–28 weeks of gestation is conducted as the diagnostic method for GDM: The blood glucose threshold of fasting, 1 h and 2 h after oral glucose is 5.1, 10.0 and 8.5 mmol/L, respectively. GDM is diagnosed when the blood glucose level reaches or exceeds the above criteria at any time point (Wei and Yang, 2018).

The procedure for the OGTT is as follows (Metzger et al., 2010): Fast for 8–10 h before the OGTT; maintain a normal diet for three consecutive days before the test, ensuring the daily intake of carbohydrates is no less than 150 g. Remain seated and refrain from smoking during the test. During the test, consume 300 mL of a liquid containing 75 g of glucose within 5 min. Collect venous blood samples before glucose intake, and 1 h and 2 h after glucose intake (time calculated from the start of glucose drinking), placing them in tubes containing sodium fluoride. Measure plasma glucose levels using the glucose oxidase method. To ensure the accuracy of the OGTT result, it is recommended to draw the fasting blood sample before 9 a.m. The evening before the OGTT, participant should avoid fasting for too long to prevent reactive hyperglycemia in the morning, which could affect the diagnosis.

### 2.2 Experimental procedures

#### 2.2.1 Acquisition of OCT images of retinal vessels

Examinations were conducted by using a Heidelberg OCTA instrument (Heidelberg Engineering, Inc., Heidelberg, Germany). The scanning light source wavelength was 870 nm; axial resolution is 12 μm; transverse resolution is 6 μm; scanning depth is 2.0 mm. The OCTA examinations for all the subjects were performed by the same highly experienced professional technician. The GDM group took OCT imaging after diagnosis, the PC group obtained OCT images through outpatient fundus screening, and the NC group used data from individuals undergoing routine health check-ups. Written informed consent was obtained from all participants for data collection.

**FIGURE 1:**
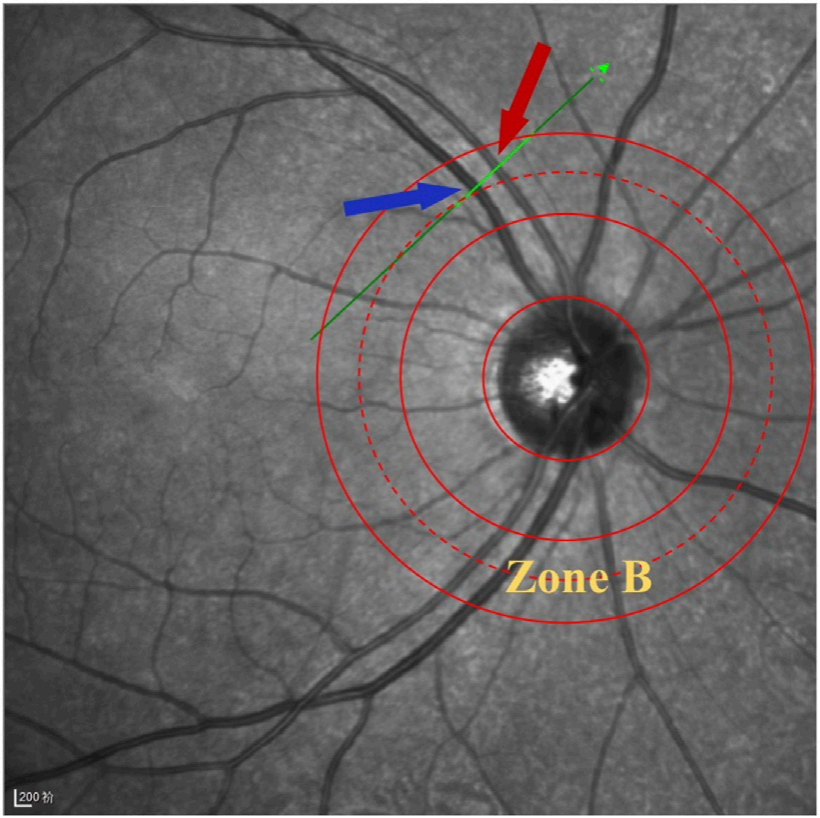
Location diagram of Zone B. The green scan line is vertical to the B zone retinal vessels, and the red arrow indicates the artery, while the blue arrow indicates the vein.

**FIGURE 2:**
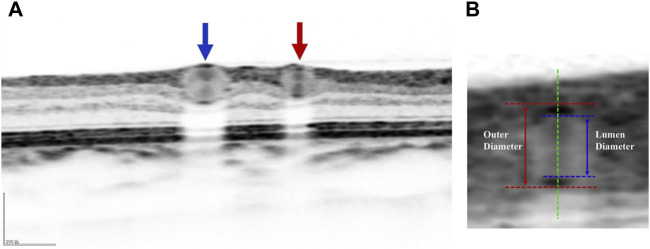
**(A)** Schematic diagram of blood vessels in retinal cross section: red arrow represents arterial cross section and blue arrows represents venous cross section. **(B)** Schematic diagram of the lumen and outer diameters of blood vessel: the red dashed line represents the outer wall of blood vessel, the blue dashed line represents the lumen diameter of blood vessel, the red solid line represents the outer diameter of blood vessel, and the blue solid line represents the lumen diameter of blood vessel.

The specific operation is as follows (Tong et al., 2015): The patient sits in front of the OCTA device, with their head resting on a chin rest and focuses on the cursor in the scanner. A specially designed positioning ring is used to locate the fundus retinal vascular scanning (Figure 1). The first determined scanning area is Zone B (Figure 1). The characteristics of the blood vessels in Zone B are more consistent with the description of small arteries and veins in morphology. Additionally, after the retinal vessels emanate from the optic disc center, there are fewer arteriovenous crossings and retinal arterial pulsation phenomena in Zone B, which do not affect the measurement of the retinal arteriovenous diameters. First, draw concentric circles on a transparent plastic film to determine Zone B. Based on the magnification of the fundus image on the OCT, the central circle is used to determine the diameter of the optic disc, and then two concentric circles are drawn with diameters of 1 and 1.5 optic disc diameters. The second and third circle diameters define Zone B. Scan the blood vessels in Zone B. The plastic film with the positioning ring is attached to the computer screen, researcher adjusted its position to be centered on the optic disc, and determined the location of the zone B. Scan the blood vessels in this area vertically. If the vessels have already branched before Zone B, then scanned the before branching. Only images that clearly display the vessel walls could be selected for further research (Figure 2). At least five clear OCT images are scanned for each vessel for analysis, excluding images with poor quality, segmentation errors, and artifacts. Measurements are taken from both eyes, but only data obtained from the right eye are used in the assessment. If imaging of the right eye is interfered by pathology or images of sufficient quality cannot be obtained, the left eye would be included for the study.

#### 2.2.2 OCT image data preprocessing

**FIGURE 3:**
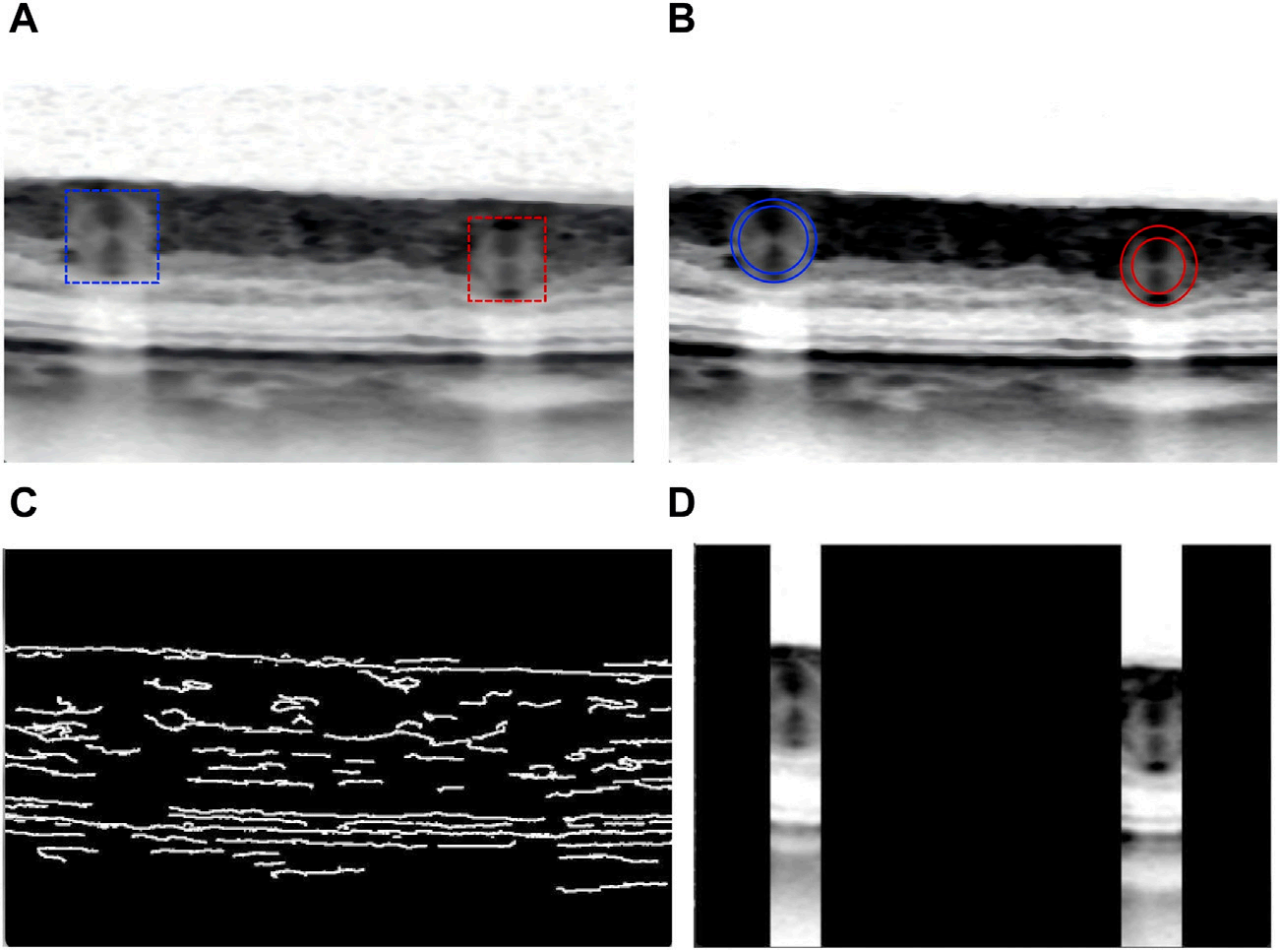
Schematic diagram of OCT image preprocessing: **(A)** Original OCT picture. **(B)** CLAHE algorithm enhances the image contrast. **(C)** Edge detection of the enhanced OCT image. **(D)** Finally generate regional ROI worthy of attention for further feature extraction.

In order to avoid unnecessary processing of unrelated structure, researchers resized the collected OCT images to a dimension of 512 × 512 pixels. Due to the low quality of fundus OCT images (Figure 3A), effective preprocessing techniques are crucial to improve image quality and accuracy. These processing methods mainly include contrast limited adaptive histogram equalization (CLAHE), canny operator edge detection, and region of interest (ROI) extraction method. CLAHE was used to improve image contrast (Sidhu et al., 2023), and the algorithm effect was shown in Figure 3B. Canny algorithm was applied to detect the edge of the enhanced OCT image, and the results were displayed in Figure 3C. Finally, ROI of noteworthy regions was generated for further feature extraction in the future, as shown in Figure 3D.

#### 2.2.3 Hyper fine image segmentation algorithm of PKSEA-Net

In this study, we introduced an innovative artificial intelligence for image recognition and processing algorithm, called PKSEA-Net, to improve segmentation accuracy by enhancing the perception of edge information in OCT images. The overall architecture of PKSEA-Net includes encoder and decoder. PKSEA-Net adopts the general architecture PVT-v2 (Wang et al., 2024) as the encoder, and the new decoder architecture is consisted of Edge-Aware Block (EAB) and pyramid feature fusion module (PFFM). EAB combines prior knowledge for supervision and multi-queries for multi-task learning, with supervised information derived from the optimized full-width-at-half-maximum (FWHM) and gradient maps. Moreover, PFFM effectively integrates multi-scale features through a novel attention fusion approach, which specifically includes the following four contents.

##### 2.2.3.1 The PVT encoder module

PVT (Pyramid Vision Transformer) is a computer vision model (Li W. et al., 2024) that combines the advantages of Transformer network and multi-scale feature pyramid. The purpose of PVT is to utilize the expression of Transformer while capturing multiple layers of visual information to obtain more accurate and robust image information. By using different size image blocks as inputs and gradually reducing the size of the image blocks at different stages of the network, feature maps of multiple resolutions are generated. These feature maps are useful for the model to comprehend and process the details of OCT images and perivascular information better.

##### 2.2.3.2 The EAB module

One of the core modules of PKSEA-Net is EAB (Hu et al., 2022), which can use the optimized FWHM algorithm to sense and detect the longitudinal edges of blood vessels, and take the results as the prior knowledge of the model to provide the supervision signal of edge information for model training. Besides, multi-query for attention information is set in EAB, so that the model can not only extract the morphology of retinal vessels, but also directly regress the diameter values of the blood vessels. Furthermore, the model designs a feature fusion module based on the attention mechanism, which can better complete the feature fusion and information extraction.

##### 2.2.3.3 The PFFM module

PKSEA-Net generates a signature signal at each scale. To make full use of multi-scale information under considering efficiency, the network incorporates these features from bottom to top. In order to enhance the perception of vascular edge information for PFFM, we use an attention fusion approach that introduces a self-attention mechanism with maintaining computational efficiency. Transformer Block (TB) is regarded as a common unit feature to transform multi-scale features (Li Z. et al., 2024), and then introduce multiplication and addition calculations to fuse them.

##### 2.2.3.4 Optimized FWHM algorithm

**FIGURE 4:**
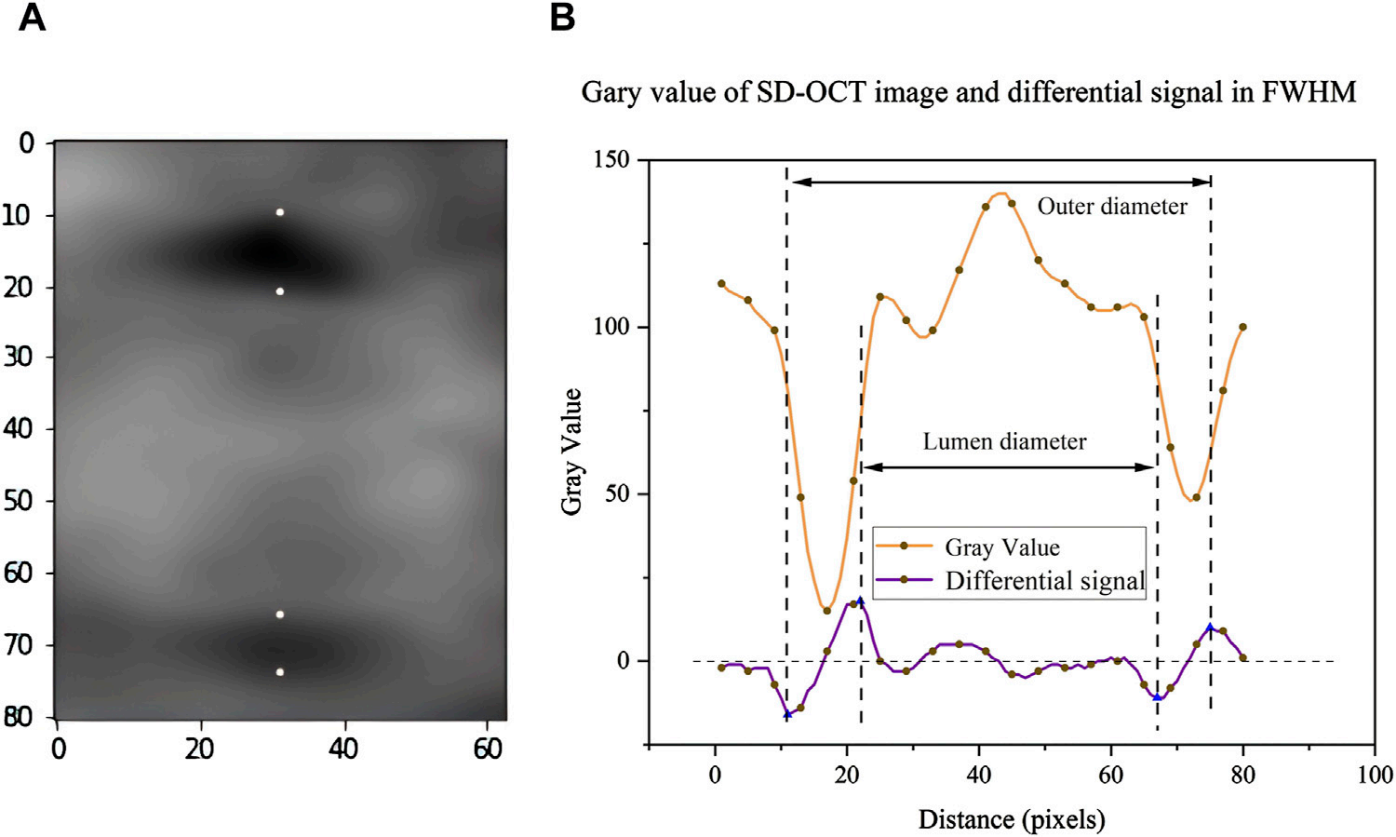
Schematic diagram of algorithm data analysis: **(A)** In the region around blood vessel in the OCT image, the four points correspond to the recognized blood vessel edges respectively. **(B)** Optimize the FWHM algorithm to obtain the corresponding gray value and its differential signal.

Previous studies (Tong et al., 2015) have already confirmed the feasibility of traditional FWHM, but this method has some errors and requires manual calculation and validation in clinical practice, so it is difficult to achieve full automation. Therefore, we propose an optimized FWHM algorithm that takes the proximal region of the blood vessel as input and scans each column of pixel sequences from left to right, within a specific range near the horizontal center of the image. This automatic scanning of candidate columns in the vascular area aims to find the most suitable solution for determining the blood vessel diameter, thereby improving the accuracy in data collecting. This method uses image processing and pattern recognition techniques to automate the measurement process, which can reduce human mistake and increase efficiency. The specific steps are as follows: at first, the original grayscale sequence is calculated by difference operation to obtain the difference sequence, which reflects the rate of change of gray values and helps to highlight the edges in the image. Next, the sequence is divided into several groups according to the zero point in the difference sequence. The zero point refers to the point with the median zero of the difference sequence, which usually corresponds to the turning point of the gray value change, indicating the edges in the image. If the first value of the difference sequence is positive and the last value is negative, then the two sequences may represent the background of the image or the non-feature regions. The groups are paired in an alternating sequence, and the points of the greatest difference between the paired positive and negative sequences are identified, which represent the edges of the blood vessels. Thus, the blood vessel region can be precisely located (Figure 4).

#### 2.2.4 Measurement of retinal vascular parameters

**FIGURE 5:**
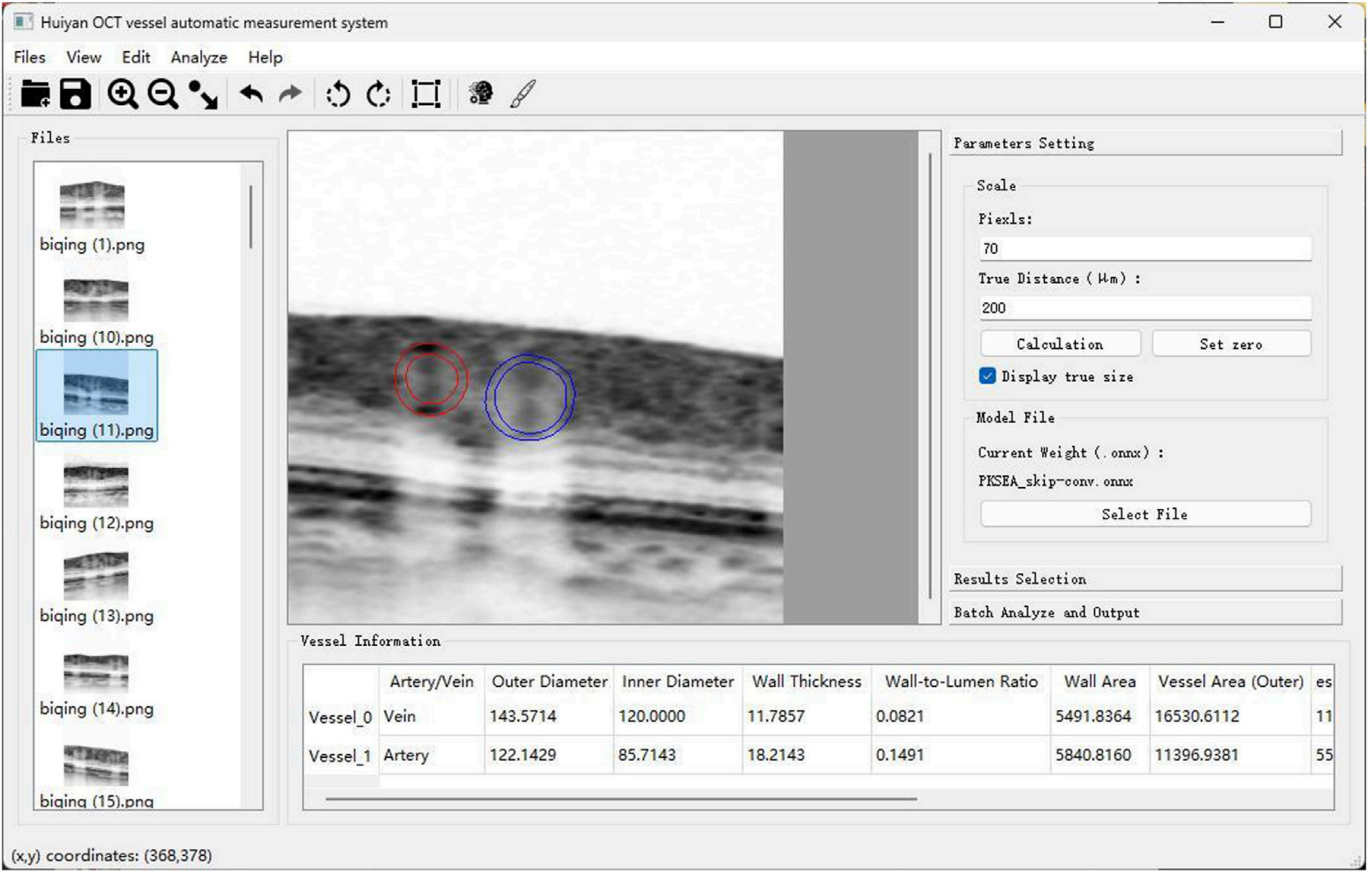
Schematic diagram of artificial intelligence blood vessel analysis software: Automatic calculation of RAOD, RVLD, RVOD, RVLD, AWT, VWT, AWLR, VWLR, AWCSA, VWCSA, and AVR.

PKSEA-Net can extract fine blood vessel edge information and accomplish high-precision measurement of blood vessel parameters, and also can strengthen the process of feature extraction through the supervision of external prior knowledge, so as to achieve the purpose of extracting and calculating relevant medical parameter information. Therefore, based on the above preprocessing technique of OCT images and PKSEA-Net, we have constructed an artificial intelligence measurement software based on deep learning. The vertical to horizontal ratio of the obtained OCT image was adjusted to 1:1 μm, and the blood vessel cross section image was saved as 512 × 512 pixels after amplification by 8 times. The image was input into this intelligent measurement software for image preprocessing (Figure 5). According to the PKSEA-Net, we calculated and analyzed retinal arteriolar lumen diameter (RALD), retinal arteriolar outer diameter (RAOD), retinal venular lumen diameter (RVLD), and retinal venular outer diameter (RVOD), arterial wall thickness (AWT) = (RAOD-RALD)/2, venular wall thickness (VWT) = (RVOD-RVLD)/2,arterial wall to lumen ratio (AWLR) = (RAOD-RALD)/2/RALD, venular wall to lumen ratio (VWLR) = (RVOD-RVLD)/2/RVLD, arterial wall cross-sectional area (AWCSA) = (RAOD^2^-RALD^2^) × 3.14/4, venular wall cross-sectional area (VWCSA) = (RVOD^2^-RVLD^2^) × 3.14/4, arteriovenous ratio (AVR) = RAOD/RVOD.

#### 2.2.5 Statistical analytical methods

SPSS 27.0 was conducted for conducting statistical analysis. If the measurement data conform to the normal distribution, it is expressed as mean ± standard deviation. One-way analysis of variance was used for comparison between groups. Least significant difference test (LSD) was used for comparison between groups with homogeneity of variance, and Games-Howell test was used for comparison between groups with uneven variance. P values < 0.05 were considered statistically significant.

## 3 Results

### 3.1 Demographics

A total of 160 subjects were included in this study, among which there were 50 in the GDM group (initially 60 were collected, but 10 were excluded due to poor image quality), 55 in the PC group, and 55 in the NC group. There was no significant difference in mean age among the three groups (*p* > 0.05), which was 31.86 ± 4.29 years old (range 23–42 years old) in the GDM group, 30.49 ± 3.53 years old (range 24–42 years old) in the PC group, and 31.22 ± 4.26 years old (range 23–40 years old) in the NC group. There was no significant difference in mean gestational age between GDM group and PC group (*p* > 0.05). In the GDM group, no one had history of prepregnancy diabetes. 2 pregnant women in GDM group received insulin injection therapy, and the other women received diet control. All subjects had no eye disease or systemic disease.

### 3.2 Measurement of retinal artery parameters

**FIGURE 6:**
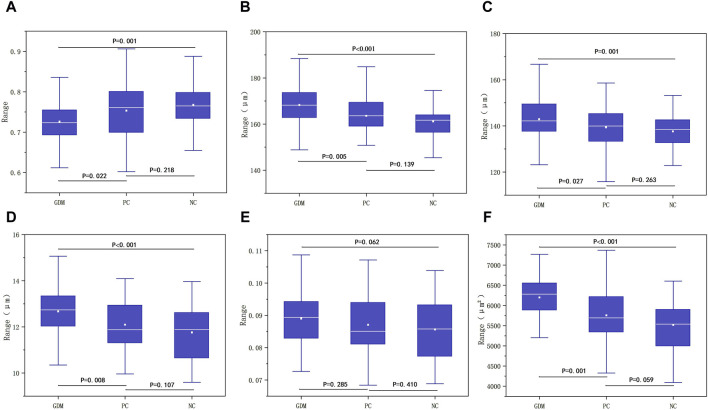
**(A)** Comparison of differences among the three groups in AVR. **(B)** Comparison of differences between the three groups in RVOD. **(C)** Comparison of differences between the three groups in RVLD. **(D)** Comparison of differences between the three groups in VWT. **(E)** Comparison of differences between the three groups in VWLR. **(F)** Comparison of differences among the three groups in VWCSA.

There were no significant differences in RAOD, RALD, AWT, AWLR and VWCSA among GDM, PC and NC groups (*P* > 0.05, Table 1). It showed the significant difference among the three groups in terms of AVR (*p* = 0.002, Table 1), and the AVR of GDM, PC and NC group were 0.7262 ± 0.0525, 0.7534 ± 0.0678, and 0.7676 ± 0.0588, respectively. It manifested a significant difference between the GDM group and the PC group (*p* = 0.022, Figure 6A), with the higher AVR in the PC group. The difference between the GDM group and the NC group was statistically significant (*p* = 0.001, Figure 6A), and the NC group had a higher AVR. There was no significant difference between the PC group and the NC group (*p* = 0.218, Figure 6A).

**TABLE 1:** Comparison of retinal artery parameters.

	GDM	PC	NC	*P* value
RAOD (μm)	122.0836 ± 10.2104	123.1346 ± 11.6011	123.6113 ± 9.6959	*P =* 0.752
RALD (μm)	94.5531 ± 10.5105	95.6198 ± 11.8401	96.9295 ± 9.1238	*P =* 0.513
AWT (μm)	13.7653 ± 1.4279	13.7574 ± 1.4343	13.3409 ± 1.6942	*P =* 0.258
AWLR	0.1478 ± 0.0250	0.1464 ± 0.0253	0.1388 ± 0.0218	*P =* 0.120
AWCSA (μm^2^)	4677.0571 ± 630.3685	4720.5964 ± 678.4434	4627.5738 ± 753.9919	*P =* 0.780
AVR	0.7262 ± 0.0525	0.7534 ± 0.0678	0.7676 ± 0.0588	*P =* 0.002

### 3.3 Measurement of retinal vein parameters

The results indicated the significant difference among the three groups in RVOD (*P* < 0.001, Table 2). The RVOD of the GDM group was 168.2741 ± 10.2512, that of the PC group was 163.5744 ± 8.0235, and 161.1570 ± 7.1684 in the NC group. The difference between the GDM group and the PC group was statistically significant (*p* = 0.005, Figure 6B), and the RVOD of the GDM group was larger. There suggested a significant difference between the GDM group and the NC group (*P* < 0.001, Figure 6B), with the higher RVOD in the GDM group. No significant difference between the PC group and the NC group (*p* = 0.139, Figure 6B).

**TABLE 2:** Comparison of retinal vein parameters.

	GDM	PC	NC	*P* value
RVOD (μm)	168.2741 ± 10.2512	163.5744 ± 8.0235	161.1570 ± 7.1684	*P* < 0.001
RVLD (μm)	142.9281 ± 9.7501	139.3810 ± 7.7640	137.6394 ± 6.7744	*P =* 0.004
VWT (μm)	12.6730 ± 0.9874	12.0967 ± 1.1046	11.7588 ± 1.1479	*P* < 0.001
VWLR	0.0890 ± 0.0084	0.0870 ± 0.0095	0.0856 ± 0.0094	*P =* 0.173
VWCSA (μm^2^)	6199.5769 ± 686.2176	5756.8380 ± 634.0372	5520.3974 ± 637.0054	*P* < 0.001
AVR	0.7262 ± 0.0525	0.7534 ± 0.0678	0.7498 ± 0.0622	*P =* 0.002

It showed that the significant difference among the three groups in RVLD (*p* = 0.004, Table 2). The RVLD of the GDM, PC, and NC groups were 142.9281 ± 9.7501, 139.3810 ± 7.7640, and 137.6394 ± 6.7744, respectively. The difference between the GDM group and the PC group was statistically significant (*p* = 0.027, Figure 6C), and the GDM group had a larger RVLD. There was a significant difference between the GDM group and the NC group (*p* = 0.001, Figure 6C), with the higher RVLD in the GDM group. There was no significant difference between the PC group and the NC group (*p* = 0.263, Figure 6C).

There were significant differences among the three groups of GDM, PC, and NC in terms of VWT (*P* < 0.001, Table 2), and the value of which in each group was 12.6730 ± 0.9874, 12.0967 ± 1.1046, and 11.7588 ± 1.1479. A significant difference was shown between the GDM group and the PC group (*p* = 0.008, Figure 6D), with the higher VWT in the GDM group. Also, it found a statistically significant between the GDM group and the NC group (*P* < 0.001, Figure 6D), and the VWT of the GDM group was larger. There was no significant difference between the PC group and the NC group (*p* = 0.107, Figure 6D).

There was no statistically significant difference in VWLR among the three groups (*p* = 0.173, Table 2), and the VWLR values of the GDM, PC, and NC group were 0.0890 ± 0.0084, 0.0870 ± 0.0095, 0.0856 ± 0.0094, respectively. The results showed no significant difference between the GDM and the PC group (*p* = 0.285, Figure 6E), no statistical significance between the GDM group and the NC group (*p* = 0.062, Figure 6E), as well as no correlation between the PC group and the NC group (*p* = 0.410, Figure 6E).

The results indicated significant differences among the three groups in VWCSA (*P* < 0.001, Table 2). The VWCSA values of the GDM group was 6199.5769 ± 686.2176, that value of the PC group was 5756.8380 ± 634.0372, and that value of the NC group was 5520.3974 ± 637.0054. There was a significant difference between the GDM group and the PC group (*p* = 0.001, Figure 6F), with the higher VWCSA in the GDM group. It also displayed a significant difference between the GDM group and the NC group, with he GDM group having a larger VWCSA (*P* < 0.001, Figure 6F). But no significant difference between the PC group and the NC group was revealed (*p* = 0.059, Figure 6F).

## 4 Analysis and discussion

GDM is a hyperglycemic state that comes out in the second and third trimester of pregnancy and is a risk factor for developing typeIIdiabetes. It can place serious health burden on both mother and baby, including the threat of pre-eclampsia, fetal growth restriction, cardiovascular disease and neonatal hypoglycemia, and even lead to embryo death (Metzger et al., 2019). Pregnant women with GDM have a risk of developing hypertensive disorders that is 1.5 times higher than healthy pregnant women (Bryson et al., 2003). It is easy to cause retinal microcirculation disorders if the GDM is without long-term control, while the changes of retinal micro-vessels are closely related to the severity of GDM and the course of diabetes (Li et al., 2017a).

Pota et al. (2024) observed the influences of pre-gestational diabetes mellitus (PGDM) and GDM on the retinal microcirculation. It discovered that compared with healthy pregnant women and the control group, both of the superficial capillary plexus (SCP) density and the deep capillary plexus (DCP) density in the GDM group decreased, indicating that GDM might give rise to alterations in the retinal microvessels. Besides, previous research also displayed (Durbin et al., 2017; Nesper et al., 2017) that with the increase in the severity of diabetic retinopathy (DR), the DCP drops sharply, and the area of the non-perfusion region in the fovea centralis of the macula increases. In contrast, Liu and Wang (2021) found that SCP density decreased significantly, while DCP density increased in GDM patients. The difference findings may be due to the redistribution of retinal vessels from shallow to deep layers during pregnancy and GDM, which could be an adaptive response of the body to hyperglycemia to protect the retina from further damage. The study by Doğan and Erkan Pota (2023) explored the effect of GDM and Preeclampsia (PIH) on the microvasculature of the retina and choroid, and found that patients with GDM complicated by PIH experienced a decrease in blood flow density and choroidal thickness. These changes might be associated with alterations in the retinal microvasculature of patients with GDM. Most of ocular changes are typically physiological and temporary during pregnancy, however, these changes might turn into severe disease without positive controls. In addition, if GDM can not be controlled properly, it may increase the risk of developing typeⅡdiabetes in pregnant women. Meanwhile, because of the similar pathophysiological abnormalities of PIH and GDM, GDM may also be a contributing factor to hypertension, causing a threat to the long-term health for pregnant women.

Due to the above-mentioned studies only concentrated on the blood flow without deep research on the level of small blood vessels. We suspect that the morphology of small retinal vessels may have changed before the abnormal blood flow density. Evliyaoglu et al. (2022) found that blood flow density in shallow and deep layers of macular fovea with GDM decreased, retinal vein diameter expanded, and AVR showed significant differences. Kifley et al. (2008) claimed that retinal artery vein diameter dilated in patients with diabetes and impaired glucose tolerance, and GDM patients may already have abnormal retinal micro-vessels during pregnancy. Although the duration is not long in the high glucose environment, it still leads to the dilation of retinal vein because of hypoxia, inflammation and endothelial dysfunction, while the retinal artery has no significant changes (Li et al., 2017a).

During pregnancy, changes in hormone levels would cause a variety of temporary physiological changes in various systems and organs throughout the body, including increases in nitric oxide levels, progesterone levels, total body fluid, and capillary hydrostatic pressure. These factors may lead to peripheral arteriolar constriction and dilation of retinal capillaries and veins (Soma-Pillay et al., 2016). However, long-term hyperglycemia and upregulation of leptin and pro-inflammatory cytokines (Chang et al., 2015) make these changes irreversible of GDM patients. Moreover, the increase of C-reactive peptide and insulin-like growth factor-1 (IGF-1) levels also had a certain effect on the progression of GDM. We analyzed the retinal vascular parameters of the GDM, PC, and NC groups in our research, and found that compared to the other two groups, the values of RVOD and RVLD increased in the GDM group, while the AVR decreased. There existed a significant difference between the GDM group and the PC group, however, no result showed any significant differences between the PC group and the NC group in the changes of retinal vascular parameters. Although the state of pregnancy may cause changes in retinal microvessels, no significant difference was observed in our study. The possible reason of these results is that pregnancy has physiological characteristics and relatively short duration, also the cardiovascular system has certain compensatory mechanisms to adapt to internal environmental changes during pregnancy.

There are few study focus on GDM and small retinal vessel morphology. Li et al. (2017b) published a cohort study that included 1,136 pregnant women in the first trimester of single pregnancy, and found a series of abnormal phenomena among GDM patients, such as narrowing of retinal arteriolar diameter, decreasing fractal dimension, and increasing branch Angle, which indicated that transient hyperglycemia during pregnancy may lead to small vessel dysfunction. However, retinal arterioles were reduced during acute hyperglycemia in type 1 diabetes mellitus (T1D) patients at the DR level (Bucca et al., 2018). Conversely, another study (Li et al., 2017c) investigated the association between T1D patients with poor blood glucose control and the retinal micro-vascular system, and after following those T1D patients for 1 year, the researcher found that patients with poor blood glucose control tended to have a wider retinal arteriolar diameter equivalent. In the early stage of diabetes or acute blood glucose rise, due to the influence of vascular endothelial dysfunction and inflammatory response, it initially leads to vascular constriction, reduced blood flow velocity, aggravated hypoxia, and narrowed retinal artery (Ikram et al., 2013). However, it will eventually cause compensatory relaxation of micro-vessels with long-term hyperglycemia. In our study, because of insufficient sample size and other reasons perhaps, we found no significant difference in retinal artery parameters between the GDM and PC groups compared with the NC groups, and subsequent studies with more GDM patients were needed.

Matuszewski et al. (2022) discovered that WT, WLR and VWCSA significantly increased by using rtx1 adaptive optical retinal camera to evaluate the morphological parameters of retinal arteriole in T1D patients. AWT is regarded as a more sensitive indicator to evaluate hypertensive retinopathy than retinal artery diameter, which could reflect the changes in the thickness of blood vessel more accurately to detect the effects of high blood pressure on retinal vessels earlier. At present, no correlation between GDM and retinal vascular wall has been reported at home and abroad. We found no significant difference in AWT among the three groups of subjects, and the VWT was larger in the GDM group. The thickening of VWT may be an important early sign of DR progression. During the development of DR, metabolic disorders and oxidative stress caused by hyperglycemia lead to changes in the structure and function of retinal micro-vessels, and such changes may also have a certain correlation with the risk of cardiovascular disease. WLR is the ratio of retinal vessel wall thickness to vessel lumen diameter and is considered to be the most accurate index to confirm retinal vessel remodeling. This index can sensitively reflect the structural changes of retinal vessels. Harazny et al. (2007) conducted the first non-invasive study of retinal vascular WLR in a group of hypertensive patients, using scanning laser Doppler flowmeter and automatic full-field perfusion imaging analysis to assess retinal arteriole structure, and found that the WLR of retinal arteriole increased with patient age. Besides, WLR can be used to identify patients treated for hypertension who are at increased risk of cerebrovascular disease. Probably, because the course of diabetes in our GDM patients is short, we did not find any difference in WLR among the three groups. However, we observed that there were significant differences in VWCSA among the three groups, with significant differences between GDM, PC, and NC group. Also, VWCSA increased in the GDM group, but no significant difference between the PC group and the NC group. Pregnancy is a natural physiological process during which the body has a certain ability to regulate, and the retinal vascular structure tends to be relatively stable, so significant pathological changes are not expected to occur. An increase in VWCSA is associated with thickening of the vascular walls, which may be due to vascular remodeling and hemodynamic changes resulting from GDM.

Nowadays, the relevant studies on retinal vascular diameter worldwide all use central retinal artery equivalent (CRAE) and central retinal vein equivalent (CRVE) to estimate the retinal vascular diameter initially. We adopted an innovative artificial intelligence technology, which is semantic segmentation based on machine deep learning, combined OCT images and PKSEA-Net to accurately locate and segment small retinal vessels, and quantitatively analyzed the outer diameter, inner diameter, tube wall thickness, wall cavity ratio, tube wall area, AVR and other parameters of the small retinal vessels. Thus, the changes of the small retinal vessels morphology can be evaluated more accurately. Meanwhile, the accuracy and efficiency of retinal vessel diameter measurement can be improved, and a new perspective can be provided for the study of retinal micro-vessel changes. Furthermore, to ensure the accuracy of the study results, we carefully controlled the confounding factors that may affect the results, excluded subjects with hypertension, hypercholesterolemia, and obesity, which are commonly associated with GDM, and selected pregnancies of similar gestational age, which allowed us to focus on a specific group of GDM patients.

It is widely known that retinal vessels are the only blood vessels in the body that can be observed directly, repeatedly and non-invasively. We used the half-peak width algorithm, whose easibility and scientificity has been verified to measure blood vessels in fundus OCT images previously. However, on account of the imaging mechanism of OCT, the ambiguity of imaging noise in OCT image is obvious. Moreover, because of the deviation of cross-section angle and the tight connection between blood vessel areas and tissues, the characteristics of retinal vessels will be randomly weakened, so it is difficult to observe clear retinal vessel boundaries, resulting in insufficient contour information around the blood vessel wall and strong subjectivity and uncertainty without fully automation. Thus, the efficiency of the measurement needs to be improved.

The boundary ambiguity has always been a common and crucial problem in medical image processing. In recent years, deep learning technology is constantly promoting the development of medical image processing, especially in the core task of image segmentation. The powerful feature extraction capability of deep learning models can be utilized to identify and segment different structures in images with greater accuracy. (Ryu et al., 2023; Wang and Dai, 2023). For instance, adopting the encoder-decoder architecture U-Net and its variants U-Net++, U-Net3+, and Attention U-Net (Zhang et al., 2023). U-Net ++ and U-Net3+ employ more complex and nested topology as well as deep supervision to capture more detailed vascular features, facilitating the learning of task-relevant features. Feature extractor based on Transformer has also been incorporated into U-Net related technology of medical image processing. The visual Transformer is employed as the encoder part and the U-Net as the decoder part, thereby combining the advantages of Transformer and U-Net.

However, owing to the low quality of OCT images, directly applying existing networks to the vessel segmentation task might result in a reduction in the accuracy of vessel segmentation and diameter prediction. The manual measurement process is able to provide some valuable prior knowledge that can intuitively guide the network to perform finer segmentation for higher segmentation accuracy. Therefore, we improved the original semi-automatic FWHM method, developed a fully automatic FWHM algorithm, and combined it with the gradient map to provide valuable edge information as prior knowledge, so as to improve the accuracy of segmentation.

Moreover, in the field of medical image processing, the prediction task of blood vessel diameter usually relied on the pixel value of the segmented output, and can only achieve the prediction at the pixel level. However, in order to achieve the precision of sub-pixel level segmentation, we applied a multi-task learning network called PKSEA-Net in this study. PKSEA-Net utilizes a multi-query attention mechanism to minimize access overhead and memory footprint by sharing partial keys and values among different attention heads. This mechanism not only precisely locates the vascular region but also acquires the vessel diameter as a regression task. We have successfully verified the accuracy and feasibility of using this technique to measure retinal vessel parameters. By measuring retinal vessels accurately, we can better understand the morphological changes of retinal vessels in GDM patients and pregnant women.

The innovation of this study is that we introduced PKSEA-Net to achieve multi-task learning, including fine segmentation of OCT images and continuous value prediction of diameters. Through enhancing the perception of edge information in retinal fundus images to improve the segmentation accuracy, identifying accurately and analyzing quantitatively of small retinal vessels, PKSEA-Net successfully solved the common medical problem of edge information ambiguity.

Some limitations still need to be improved in the further research in our study. Firstly, our study selected cross-sectional data that only can reflect the situation at a point in time, which can not show the trends over time. Secondly, the small sample size might have limited the capacity of finding minor differences. Thirdly, the morphological changes of retinal vessels are a long-term process, which requires in-depth analysis through follow-up for a long time, especially for the quantitative analysis of retinal artery parameters, which requires more data of GDM patients to support these findings. In addition, the course of GDM is slightly different among individuals, which may show impacts on the study results. To assess the effects of GDM on retinal vessels more accurately, future research will further expand the sample size, collect more data from patients with GDM, and include the *postpartum* population for case-control studies.

## 5 Conclusion

In conclusion, although GDM is recognized as a temporary metabolic disorder, we observed that there were increases in RVOD, RVLD, VWT, and VWCSA, decrease in AVR in patients with GDM. However, no significant difference of retinal vascular parameters was shown between normal pregnant women and normal non-pregnant women. These findings indicated that even though the GDM have not been clinically confirmed, PKSEA-Net technology can assist to identify changes in retinal vascular morphology and diagnose micro-vascular lesion early in normal pregnant women and high-risk groups of GDM.

## Data Availability

The raw data supporting the conclusions of this article will be made available by the authors, without undue reservation.
